# Unlocking the Potential of 46 New Bacteriophages for Biocontrol of *Dickeya Solani*

**DOI:** 10.3390/v10110621

**Published:** 2018-11-10

**Authors:** Alexander B. Carstens, Amaru M. Djurhuus, Witold Kot, Deborah Jacobs-Sera, Graham F. Hatfull, Lars H. Hansen

**Affiliations:** 1 Department of Environmental Science, Aarhus University, Frederiksborgvej 399, 4000 Roskilde, Denmark; aca@envs.au.dk (A.B.C.); amma@envs.au.dk (A.M.D.); wk@envs.au.dk (W.K.); 2 Department of Biological Sciences, University of Pittsburgh, Pittsburgh, PA 15260, USA; djs@pitt.edu (D.J.-S.); gfh@pitt.edu (G.F.H.)

**Keywords:** soft rot Enterobacteriaceae, SRE, *Dickeya solani*, potato, biocontrol, phage therapy, phage biocontrol, phage cocktail, soft rot, blackleg

## Abstract

Modern agriculture is expected to face an increasing global demand for food while also needing to comply with higher sustainability standards. Therefore, control of crop pathogens requires new, green alternatives to current methods. Potatoes are susceptible to several bacterial diseases, with infections by soft rot Enterobacteriaceae (SRE) being a significant contributor to the major annual losses. As there are currently no efficient ways of combating SRE, we sought to develop an approach that could easily be incorporated into the potato production pipeline. To this end, 46 phages infecting the emerging potato pathogen *Dickeya solani* were isolated and thoroughly characterized. The 46 isolated phages were grouped into three different groups based on DNA similarity, representing two distinct clusters and a singleton. One cluster showed similarity to phages previously used to successfully treat soft rot in potatoes, whereas the remaining phages were novel and showed only very limited similarity to previously isolated phages. We selected six diverse phages in order to create the hereto most complex phage cocktail against SRE. The cocktail was applied in a proof-of-principle experiment to treat soft rot in potatoes under simulated storage conditions. We show that the phage cocktail was able to significantly reduce the incidence of soft rot as well as disease severity after 5 days of storage post-infection with *Dickeya solani*. This confirms results from previous studies that phages represent promising biocontrol agents against SRE infection in potato.

## 1. Introduction

Soft rot *Enterobacteriaceae* (SRE) are plant pathogenic, gram-negative bacteria of the *Pectobacterium* and *Dickeya* genera (formerly *Erwinia carotovorum* and *Erwinia chrysanthemi*, respectively). Collectively, bacteria classified as SRE cause soft rot in up to 50% of angiosperm plant orders, including many economically important fruit, vegetable and ornamental crops [1]. Bacterial soft rot is considered one of the most destructive bacterial diseases in agriculture, with an estimated 15–30% of crop production being affected [2]. In potatoes, SRE are the causative agents of blackleg and soft rot diseases, affecting crop production in both the field during the growing season [3,4] and post-harvest during storage of potato tubers, where SRE have been characterized as the most problematic pathogens [5].

Historically, *Pectobacterium sp.* have been the most problematic for potato harvests in temperate regions [6]. However, recently a novel SRE pathogen of potato, *Dickeya solani (D. solani)*, has been identified. Since its discovery *D. solani* has been causing increasingly larger problems for crop production in large parts of Europe [6]. Interestingly, the strains of *D. solani* isolated from potatoes have so far been shown to be genetically closely related, even when isolated from geographically distant locations, indicating very limited diversity within the group [7,8]. This presents a promising opportunity for the use of phage biocontrol, where the narrow host range of phages is generally viewed as one of the drawbacks in a therapeutic context. Furthermore, phage biocontrol has previously been used to control SRE in potatoes with some success, both in whole tubers [9,10,11] (manuscript submitted to FEMS Microbiology Letters thematic Issue on Microbial Food and Feed Ingredients), potato plants [12] and in field trials [10]. Some of these have been performed on *Pectobacterium* species [9,11] (manuscript submitted to FEMS Microbiology Letters thematic Issue on Microbial Food and Feed Ingredients), while others have focused on the emerging pathogen *D. solani*. All studies performed on *D. solani* used simple phage cocktails containing only one or two phages [9,10,12]. All phages used in these experiments are related to the first sequenced dickeya phage vB_DsoM_LIMEstone1 [9,10,12], similar to the cluster B phages described here. Even though the authors did see a positive effect of phage treatment in these experiments, the effect was not significant at a lower phage concentration and could not be replicated in field trials [10]. We sought to increase the diversity of phages that are included in a phage cocktail in order to decrease the chance that the pathogen manages to evade phage infection and thereby increase the effectiveness of phage treatment. We therefore isolated 46 new *D. solani* phages. The isolated phages exhibit a large degree of genetic variation, forming three distinct groups, two of which have no close relatives in the publicly available databases. Furthermore, we show that a cocktail containing six of the isolated phages is able to reduce the incidence of soft rot caused by *D. solani* in whole potato tubers under simulated storage conditions.

When using phages in a biocontrol context, it is important to consider the biotic and abiotic factors known to affect phage stability [13,14]. Applying phages post-harvest could alleviate some of these effects, such as exposure to UV radiation and desiccation, while still reducing spoilage by limiting the spread of disease from latently infected potato tubers within the confined space of a storage unit. We therefore tested the phage cocktails’ tolerance towards relevant stresses such as freezing and UV radiation.

## 2. Materials and Methods

### 2.1. Phage Isolation

All incubations were performed at room temperature in lysogeny broth (LB) supplemented with CaCl_2_ and MgCl_2_ to a final concentration of 10mM. For LB plates, 15 g/L agar was added, and for top agarose, 6 g/L of agarose was added. The phages were isolated from waste water and organic waste (Table S1) using *D. solani* (DSM 28711) as a host. Phages were isolated both using direct plating and through enrichment cultures. Single plaques were purified three times to obtain a pure phage culture. To obtain high phage titers, phage high titer cultures were purified by PEG purification, as described elsewhere [15].

### 2.2. DNA Isolation and Sequencing

Phage DNA was isolated from a high titer phage suspension. Briefly, 200 µL phage lysate was added to sterile 1.5 mL Eppendorf tubes. 5U of DNase I was added, followed by incubation at 37 °C for 1 h. Subsequently, 1% SDS was added to a final concentration of 0.1%. Following this, 6U (10 µL) Proteinase K was added to the solution, followed by incubation at 55 °C for 1 h followed by an additional incubation step at 70 °C for 10 min to deactivate the proteinase. The DNA was then purified and concentrated using a DNA Clean & Concentrator™-5 kit (Zymo Research, Irvine, CA USA) according to manufacturer’s protocol and eluted with 20 µL elution buffer. Sequencing libraries were build and sequenced, as previously described [16].

### 2.3. Assembly

Assembly was performed in CLC Genomic Workbench 10.1.1 (CLC bio, Aarhus, Denmark). Briefly, overlapping reads were merged using the merge overlapping pairs tool with the following settings: gap cost, 3; mismatch cost, 2; maximum unaligned end mismatches, 0; minimum score, 8. The reads were then trimmed using standard settings and an added trim adaptor list with potentially contaminating sequencing adapters and indexing barcodes from the Nextera™ kits and assembled de novo using a de novo assembly tool. The assembly was cross-verified by SPAdes Genome Assembler version 3.10.0 [17]. Sequences from phage DNA were screened for contaminating sequencing adapters and indexing barcodes, as described earlier [16]. Cleaned Illumina reads were assembled using SPAdes with the “careful” option enabled.

### 2.4. Annotation

Open reading frames were identified in DNA Master version 5.23.2 [18] using GeneMark4.3 [19] and Glimmer version 3.02 [20] with favoring Genemark in conflicting genes. Open reading frames were hereafter manually curated. Functions were assigned using BlastP against all non-redundant protein sequences [21] and with HHpred using the databases: PDB, SCOP, Pfam, NCBI_CONSERVED_Domains. tRNAs were identified using tRNAscan-SE [22] and ARAGORN [23]. The start position of terminally redundant phages was moved to mimic that of the related phages. The genomes were uploaded to GenBank with accession numbers MH059639 (Ninurta), MH059632 (Dagda), MH807810 (Dagda_B1), MH807813 (Katbat), MH807815 (Luksen), MH807817 (Mysterion), MH807812 (Kamild) MH807820 (Coodle).

### 2.5. Comparative Genomics

Comparison of nucleotide sequences between isolated phages was performed using BLASTn [21]. All vs. all nucleotide comparisons were made using Gegenees version 2.2.1 [24], which was preformed using the BLASTn algorithm, with a Fragment size of 50, a step size of 25, and a threshold of 0%. Genomic maps were created using Phamerator version 236 [25], employing a custom database containing the phages Mysterion, Dagda, Dagda_B1, Katbat, Luksen, Ninurta and enterobacteria phage T7 (NC_001604). Multiple Sequence Alignment of conserved proteins was performed in MEGA X [26] using MUSCLE. Phylograms based on conserved proteins were made using MEGA X [26], using the Maximum likelihood method with the Nearest neighbor interchange heuristic method, and using the Jones Taylor Thornton substitution model. The robustness of the resulting trees was evaluated by 500 bootstrap replicates. Phylogeny analysis was conducted with VICTOR using nucleotide input [27].

### 2.6. Phage Stability

The six phages included in the cocktail (Dagda, Mysterion, Luksen, Coodle, Kamild, Ninurta) were treated in parallel. PEG purified phage solutions were diluted to an appropriate titer, ~10^8^ PFU/mL, and exposed to different treatments. Freezing: 20 µL of the phage stocks was diluted in 180 µL SM buffer. The samples were exposed to three freeze thaw cycles of 2 h at −18 °C followed by thawing and repeated freezing. Chloroform: 20 µL of the phage stocks was mixed with SM buffer and chloroform to a final concentration of 20% chloroform and incubated for at least 2 h at room temperature. Ethanol: 20 µL phage stock was added to 140 µL SM buffer and mixed with 40 µL ethanol to a final concentration of ~19.6% ethanol and incubated for 2 h at room temperature. UV radiation: 500 µL of the phage stock was pipetted onto the lid of a sterile Petri dish and exposed to UV (OSRAM PURITEC UV-C Germicidal Low Pressure Lamp, HNS 15W G13 G15T8/OF, at a distance of approximately 50 cm from the plates, approximately 200 µW/cm^2^) for 45 seconds before collecting the UV treated sample with a pipette. All the treatments were then added to micro titer plates and diluted in a 10-fold serial dilution in SM buffer and 5 µL of the dilutions 10^0^–10^−7^ were spotted onto LB plates, which had been prepared using 9 mL of soft LB and 250 µL of overnight culture with the appropriate host. Each assay was performed in parallel using three technical triplicates.

### 2.7. Potato Infection Model

The in planta phage treatment was based on a tuber maceration assay previously described by Dees et al. [28]. Prior to the assay, *D. solani* was streaked onto LB agar plates and incubated for 48 h at room temperature. Bacteria from these plates were then scraped from the plates and suspended in sterile distilled water and adjusted to OD A_600_ = 1.0 ± 0.2 (approximately 1 × 10^9^ CFU/mL). Ware potato tubers of the cultivar Ditta were surface-disinfected using 1% sodium hypochlorite for 15 min followed by rinsing in sterile water and subsequently air-dried at room temperature overnight in a flow hood. Each group of potatoes (*N* = 45) was then wounded using a sterile steel rod (diameter: 2 mm, depth: 10 mm) and subsequently washed in either a phage solution, containing approximately 5 × 10^8^ PFU/mL of each of the six selected phages (Dagda, Mysterion, Luksen, Coodle, Kamild, Ninurta) in 200 mL of SM buffer, or sterile water. The potatoes were then inoculated with 10 µL of bacterial suspension and sealed with white Vaseline and parafilm. The tubers were kept in plastic boxes with lids out of direct sunlight for 5 days at room temperature. After incubation, the potatoes were cut through the inoculation point. The macerated tissue was removed using a spatula and the amount of lost tissue was quantified by measuring the weight of the potatoes before and after removal of the macerated tissue.

## 3. Results and Discussion

### 3.1. Phage Characterization

A total of 46 phages infecting *D. solani* were isolated from a variety of sources (Table S1). Based on DNA homology, the phages can be clustered into three different groups with less than 0.2% DNA sequence similarity between groups and more than 48% DNA sequence similarity inside each group (Figure S1). The groups will hereafter be referred to as Cluster A and B as well as the singleton and Ninurta.

**Figure 1:**
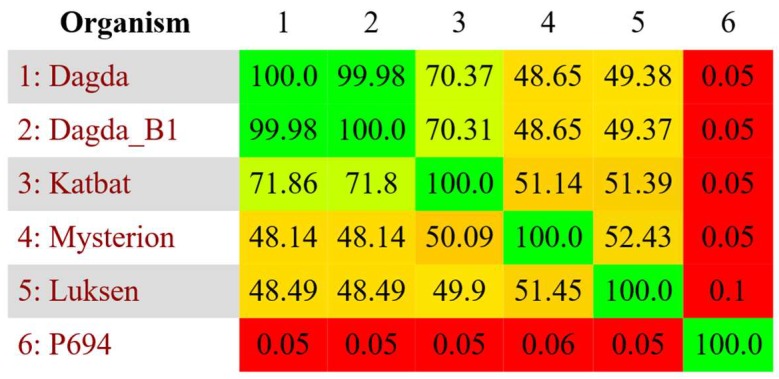
All vs. all nucleotide similarity analysis on all the Cluster A phages with escherichia phage P694 (KP090454) included as a reference, made using Gegenees [24].

**Figure 2:**
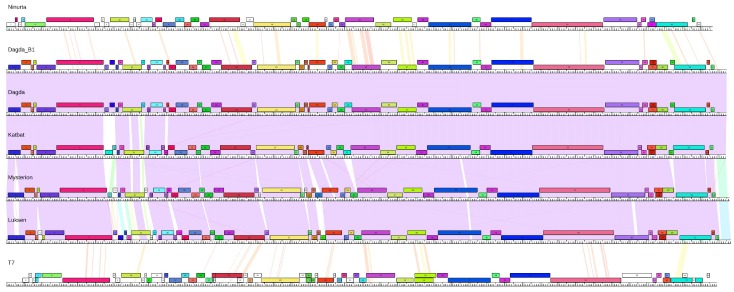
Phamerator map [25]: Pairwise alignment of Mysterion, Dagda, Dagda_B1 Katbat, Luksen and Ninurta with the genome of Enterobacteria phage T7 (NC_001604) included as a reference. Each genome is represented by a vertical line with intersecting colored lines representing nucleotide similarity, red being more dissimilar and violet being more similar. Colored boxes represent genes.

Cluster A contains five phages, which have a genome size between 40,745 bp (Katbat) and 41,133 bp (Luksen), and have a GC content of 48.0–48.2%. This is lower than the 56.3% found in the host, which is common amongst phages [29]. No close relatives of the Cluster A phages exist in the publicly available databases as determined by BLASTn, but the closest relative is the T7-like escherichia phage P694(KP090454) [30] which shares less than 1% sequence similarity with any Cluster A phages (Figure 1). Cluster A phages are quite diverse, with the individual phages sharing only between 48.5% and 71.9% sequence similarity. The exceptions are Dagda and Dagda_B1, which only differ by four base pair substitutions (Figure 1, Figure S1). Despite very low nucleotide similarity, the Cluster A phages share many genes, as well as an overall genome synteny, similar to that found in the T7-like phages (Figure 2). This includes: head-to-tail connector protein, capsid assembly protein, major capsid protein, tail tubular protein A, tail tubular protein B, internal virion protein A, internal virion protein B, internal virion protein C, internal virion protein D, tail fiber protein, small subunit terminase, large subunit terminase and a type II holin. The phages also contain a gene pair with homology to Rz lysis protein and Rz1 lysis protein that have a large overlap in their reading frames [31] (Figure 2). Furthermore, the phages also contain a single subunit RNA polymerase, a hallmark of the *Autographivirinae* subfamily [32]. Besides this core “T7-like genome”, the phages contain a high number of genes with unknown function. Approximately half (42–49%) of the genes in the Cluster A genomes could not be assigned a function. No tRNAs were identified in any of the Cluster A phages. Analysis of the conserved proteins indicate that Cluster A phages internally are quite similar but are distinct from their closest relatives (Figure S2). Taken together with whole genome comparison of the cluster A phages and their closest relatives (Figures S3 and S4), as well as the conserved structure of the cluster A phages (Figure 2), this indicates that the Cluster A phages might represent a new novel genus in the *Autographivirinae* subfamily.

Cluster B is the largest cluster, comprising 40 phages. The genome sizes of Cluster B phages are rather large (~150kb) and have a GC% content ranging from 49.0% to 49.4%. A query in publicly available databases revealed several phages that are closely related to Cluster B phages, including dickeya phage vB_DsoM_LIMEstone1(HE600015). LIMEstone1 has already shown promise in the control of *D. solani* in potatoes in vivo [10]. The Cluster B phages share a genome structure vaguely similar to that of the T4-like phages, and they all contain a single tRNA with the anticodon 5’-CAT. Cluster B phages also contain an unusually large number of homing endonucleases, with 18 in phage Kamild, corresponding to 10.8% of all nucleotides in the entire genome encode endonucleases. This is similar to the 15 homing endonucleases found in T4, although this is not a general characteristic of the T4-like phages [33]. These homing endonucleases could be seen as genetic parasites, simply ensuring their own replication at the cost of the phage. However, how these affect phage lifestyle is unclear. It has been shown that the homing endonucleases increase the horizontal gene transfer of surrounding genes when two related phages are co-infecting the same host [34,35]. Although some homing endonucleases in Cluster B pages appear to be conserved, others contain deletions or frameshift mutations. Still others are only found in some members of the cluster, indicating that they have either recently been acquired or have been lost in other phages. Cluster B phages appear to be fairly widespread in the environments tested, constituting 40 out of the 46 phages isolated. Even though Cluster B contains a considerably higher number of phages, the Cluster B phages are much less diverse than the Cluster A phages, having pairwise nucleotide similarities above 69% within the cluster. Many of the Cluster B phages are closely related, sharing above 90% sequence similarity (Figure S1). However, two distinct sub clusters exist within Cluster B (blue squares in Figure S1) with lower similarity between sub clusters. We therefore chose to focus on one phage in each sub cluster (Kamild and Coodle). Based on the high degree of sequence similarity between cluster B phages and Limestone1 (BLASTn), as well as their conserved genome structure (Figure S5), we suggest including the cluster B phages in the *Limestonevirus* genera.

Ninurta has a genome size of 40286 bp and has only one close relative in the publicly available databases; the recently sequenced dickeya phage vB_DsoP_JA10 (MH460459.1). Besides JA10, Ninurta share some sequence similarities with a number of T7-like phages, with the highest similarity being to pectobacterium phage PP74 (KY084243.1) with a query coverage of 60% and 73% nucleotide identity (BLASTn). Despite sharing less than 0.2% nucleotide sequence similarity to any of the Cluster A phages, Ninurta has a T7-like genome structure, similar to the Cluster A phages (Figure 2). This structure includes genes encoding: head-to-tail connector protein, capsid assembly protein, major capsid protein, tail tubular protein A and B, internal virion protein A–D, tail fiber protein, type II holin, small subunit terminase, large subunit terminase, Rz/Rz1 lysis proteins as well as a single subunit RNA polymerase. Ninurta has a GC content of 51.0%. This is higher than that of any of the other phages isolated but still more than 5% lower than that of the host. Analysis of the conserved proteins (Figure S2), as well as a nucleotide comparison between its closest relatives (Figures S3 and S4), reveal that Ninurta and JA10 constitute a cluster of phages distinct from its closest relatives. Indicating that Ninurta and JA10 might represent a novel genus in the *Autographivirinae* subfamily.

### 3.2. Formation of Phage Cocktail

We subsequently selected a subset of phages to be included in a phage cocktail for the later treatment of SRE-infected potatoes to test the efficacy of the phages for biocontrol of soft rot under in vivo conditions. Of the 46 sequenced phages, six phages (Dagda, Mysterion, Luksen, Coodle, Kamild, Ninurta) were chosen. Diverse phages were chosen to minimize the risk that a host develops resistance towards all phages in the cocktail, therefore, phages from each cluster were included. Preference was also given to phages that produced higher titers in liquid cultures, in order to ease phage production.

All six phages gave rise to clear plaques under the conditions tested, indicating that the phages are virulent. This is supported by the genetic data, as none of the six phages contain the genes typically associated with temperate phages, such as integrases, and because they have genome organizations similar to that of other strictly virulent phages. It has previously been suggested that the use of temperate phages in phage therapy should be limited in order to lower the risk of horizontal gene transfer [36,37].

The genomes of the six phages were all manually inspected for the presence of genes with similarity to known toxin or virulence genes, and none were identified. However, since fewer than half of the genes in the six phages could be ascribed a function, it is possible that unknown virulence or toxin genes remain undetected.

### 3.3. Phage Stability

**Figure 3:**
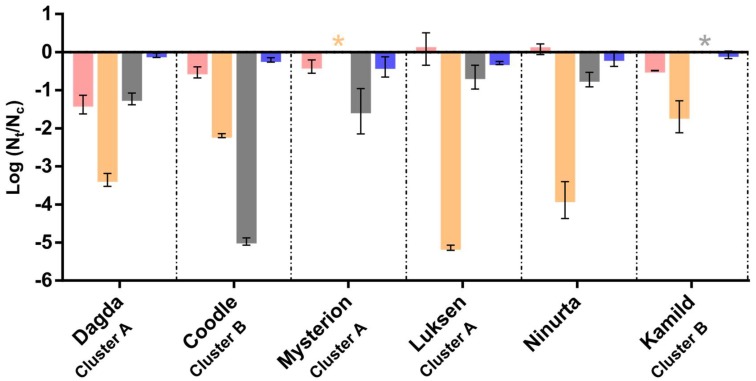
Stability assay: Effect of 20% ethanol (red), UV radiation (orange), three freeze/thaw cycles (grey) and chloroform (blue) in vitro on the stability of six phages used in the phage cocktail. Values indicate the titer post exposure relative to the control. Error bars indicate ± SD. Asterisks indicate titers below the detection limit.

To test phage stability under conditions relevant for the production, preservation and application of the phages, we conducted assays with chloroform, ethanol, UV exposure and freeze and thaw cycles. All phages were resistant to chloroform (Figure 3). However, whilst chloroform represents a useful tool to ensure the removal of host cells from the phage cultures in a laboratory setting, its toxicity makes it unsafe for application in food products. Nonetheless, all phages tested showed less than a 1 log reduction in titer after treatment with 20% ethanol, with the exception of Dagda, which had a 1.6 ± 0.25 log reduction in titer (Figure 3). This opens up the use of ethanol as a cheap and safe alternative to ensure the removal of host cells, while also possibly preserving the phage particles prior to application. If ethanol is applied directly to the phage stock during production, the concentration of ethanol should not negatively affect potato tubers, as the ethanol would evaporate during the drying process. This needs to be further tested in vivo.

As expected, all six phages were shown to be sensitive to UV radiation (Figure 3), although the degree of sensitivity varied greatly. Where the three Cluster A phages and Ninurta all showed a more than 3 log reduction, the two Cluster B phages tested, Coodle and Kamild, showed only a 2.2 ± 0.05 and 1.69 ± 0.42 log reduction, respectively. This indicates that the two phages have at least a partial tolerance to UV radiation. Interestingly, the two phages that show tolerance to UV radiation are very sensitive to freeze-thaw cycles, with Coodle experiencing a >5 log reduction in titer and Kamild dropping below the detection limit (>6 log reduction in titer). This is in contrast to the only 0.72 ± 0.19 to 1.55 ± 0.6 log reduction observed for the other phages. The phages sensitivity to UV makes treatment of the above parts of plants undesirable, we therefore focused on treatment of potato tubers. The differential response to stress and storage conditions emphasizes the usefulness of complex multi-phage cocktails to increase both robustness and modes of action under different conditions.

### 3.4. Potato Infection Model

**Figure 4:**
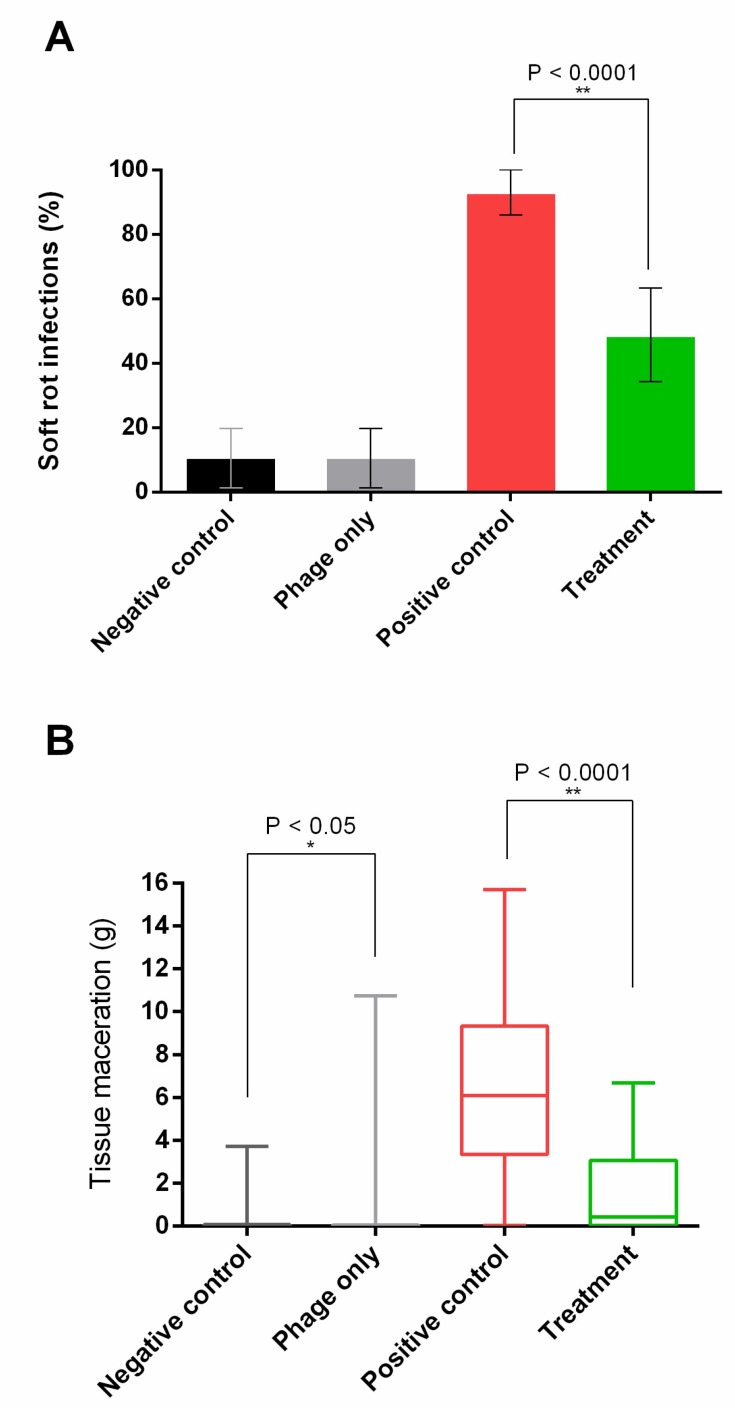
Potato maceration assay: Negative control (black); potatoes not exposed to neither phages nor bacteria. Phage only (grey); Potatoes washed in phage cocktail solution, but not exposed to bacteria. Positive control (red); Potatoes washed in sterile water and inoculated with bacteria. Treatment (green); Potatoes washed in phage solution and inoculated with bacteria. *N* = 45 for each treatment group. (**A**) Soft rot disease incidence (%): Positive soft rot infection was identified as the presence of >0.5 g macerated potato tissue. Numbers indicate the percentage of infected potatoes; error bars show 95% confidence interval. The treatment group showed a significant reduction compared to the untreated group (Chi-squared test, *P* < 0.0001). (**B)** Box plot showing the amount of macerated tissue (g) of potatoes after 5 days incubation.

Phage treatment was shown to significantly lower both disease incidence, as indicated by >0.5 g macerated tissue 5 days post-infection (Figure 4a), and disease severity (*P* < 0.00001, Mann-Whitney U Test) (Figure 4b). The negative control and the treatment control had an identical disease incidence of 11.1% (5 tubers). The presence of diseased potatoes in the controls, where no bacteria were added, is most likely caused by native bacteria or fungi infecting the wet and wounded potatoes, as we used non-sterile ware potato tubers. However, cross contamination from the other samples cannot be ruled out. The average amount of diseased tissue was slightly higher in the treatment control compared to the negative control (0.55 g and 0.32 g, respectively). However, since the data is not normally distributed and the absolute amount of diseased tissue is very low in most tubers (median of the treatment control is (0.04 g), individual tubers can have a disproportionate impact on the average. In fact, a Mann-Whitney U Test reveals that disease severity was significantly lower in the treatment control compared to the negative control (*P* < 0.05). Indicating that phage treatment has a positive effect on potato tubers, even when no bacteria are present, possibly by removing native soft rot bacteria. However, it should be noted that although the results are significant at a 5% confidence interval, it is not significant at a 1% confidence interval. In this experiment, a high concentration (approximately 1 × 10^9^ CFU/mL) of *D. solani* was added to an open wound; these conditions greatly favor bacterial reproduction and disease development, and are not unlike conditions naturally occurring during potato production, where potatoes can be wounded at several stages, especially during harvest or planting. Our results indicate that the phage cocktail used effectively reduces soft rot in wounded potatoes under storage conditions, which could potentially also reduce the incidence of soft rot and blackleg by treating seed tubers at planting. It is unknown how well the phage cocktail removes *D. solani* from latently infected potatoes, where the concentration of bacteria, as well as their growth rate, is expected to be considerably lower. It is, however, well known that phage efficiency is connected to host abundance. As such, lower concentrations of host bacteria actually might prove harder to treat using phage biocontrol, as the bacterial population might be too small to support phage replication.

We chose to run our experiment for 5 days, which is longer than the 3 days used in other soft rot phage therapy trials on potatoes [10,11]. Nevertheless, it is possible that further increasing incubation time would result in more infected tubers, either by the cells becoming resistant to the phages used or by other means of evading phage infection.

As previously described, *D. solani* has recently emerged as a pathogen of potatoes with very low genetic diversity [7,8]. Because of the low diversity of this newly emerged pathogen, we hypothesize that phages that infect the strain of *D. solani* used in this experiment (DSM 28711) will also be able to infect all or most other *D. solani* strains causing soft rot disease in potatoes. However, this hypothesis still needs to be tested.

Although phage treatment lowers the disease incidence in infected tubers from 93.3% to 48.9% (Figure 4A), almost half of the tubers show some soft rot symptoms, all be it at a significantly reduced severity (Figure 4B). Therefore, although phage treatment effectively reduced soft rot symptoms, it failed to completely eliminate the infection from all tubers. It is unclear if the bacteria that manage to establish an infection in phage-treated tubers become resistant to all of the phages in the cocktail or how they otherwise evade phage infection. A genetically diverse set of phages was used for the phage cocktail in order to lower the probability that a bacterium will develop resistance towards all phages in the cocktail. However, the possibility of cross-resistances emerging to all phages in the cocktail has not been tested and can therefore not be ruled out. Further studies are needed to determine whether the emergence of cross-resistance might limit the effect of phage biocontrol treatment. Even though phage treatment did not completely eliminate soft rot symptoms after *D. solani* infection, it managed to reduce the amount of diseased tissue by 75.3% (Figure 4B). Taken together with previous studies on phage treatment of *D. solani* [9,10,12] and *P. Atrosepticum* [11] (manuscript submitted to FEMS Microbiology Letters thematic Issue on Microbial Food and Feed Ingredients), this indicates that phage biocontrol can be used to effectively control SRE in potato tubers. However, further research is needed to determine the factors that prevent complete elimination of the pathogen so that steps can be introduced to further increase the effectiveness of such treatments.

## 4. Conclusions

Out of the 46 isolated phages, 40 of them belong to Cluster B. The isolated Cluster B phages are genetically similar but cluster into two distinct sub clusters (Figure S1). These phages were isolated from different environments, at different time points and at different locations in Denmark, indicating that Cluster B phages are both widespread and numerous. The remaining six phages all share a genome organization similar to that found in the T7-like phages. However, despite the similar genome structure, the phages have very limited nucleotide similarity (Figure S1) and might therefore represent two novel genera according to ICTV guidelines [38]. Six of the isolated phages were included in a phage cocktail and applied in an in vivo experiment on potato tubers. The phage cocktail reduced both disease severity and disease incidence, indicating that phage biocontrol could be a promising alternative method for combating soft rot. The authors suggest introducing phage biocontrol at two places in the potato production pipeline: either after harvest to reduce spoilage caused by soft rot during storage or immediately prior to planting to reduce both soft rot and potentially blackleg during the growing season. It still needs to be investigated whether phage biocontrol can efficiently remove *D. solani* from latently infected potatoes, where the bacterial load can be very low. For the closely related SRE, *P. atrosepticum,* blackleg is unlikely to develop if the bacterial load is lower than 10^3^ CFU per tuber [39]. However, it is unknown whether phage treatment can effectively lower the bacterial load to these levels. However, a smaller reduction in bacterial load will also be expected to lower disease severity and disease incidence. If successful, this could be very useful for seed producers to ensure seeds are free of *D. solani*. Moreover, using phage biocontrol might assist in halting the rapid spread of this emerging pathogen.

## Supplementary Materials

The following are available online at http://www.mdpi.com/1999-4915/10/11/621/s1; Figure S1: All vs all nucleotide comparison between all isolated phages; Figure S2: Phylogenetic analyses of amino acid sequences from three conserved proteins. A) Major capsid protein; B) RNA polymerase; C) Terminase, large subunit. The sequences are derived from Cluster A phages and Ninurta with the two closest relatives to each included for comparison (as determined by BLASTn); yersinia phage YpP-G (JQ965702), escherichia phage P694 (KP090454), pectobacterium phage PP74 (KY084243), dickeya phage vB_DsoP_JA10 (MH460459). The sequences of Dagda and Dagda_B1 are identical and therefore Dagda_B1 is excluded from the analysis. The bootstrap percentages are shown next to each node, branch lengths measured in the number of substitutions per site; Figure S3: All vs all nucleotide comparison between Cluster A phages and Ninurta, with the two closest relatives to each included for comparison (as determined by BLASTn) YpP-G (JQ965702) P694 (KP090454) PP74 (KY084243) vB_DsoP_JA10 (MH460459); Figure S4: VICTOR-generated phylogenomic analysis of Cluster A phages and Ninurta, with the two closest relatives to each included for comparison, performed using the distance formula D0. Values derived from 100 Pseudo-bootstrap replicates are shown at branches; Figure S5: Phamerator map showing the genomes of Coodle and Kamild with dickeya phage vB_DsoM_LIMEstone1 (NC_019925.1) included as a reference. Each genome is represented by a vertical line with intersecting colored lines representing nucleotide similarity, red being more dissimilar and violet being more similar. Colored boxes represent genes; Table S1: Isolation source of the 46 isolated phages.
