# Supplementary figures and images for: Unlocking the Potential of 46 New Bacteriophages for Biocontrol of Dickeya Solani
Source: Viruses. 2018 Nov 10;10(11):621. doi: 10.3390/v10110621 (PMC6267328; doi:10.3390/v10110621)

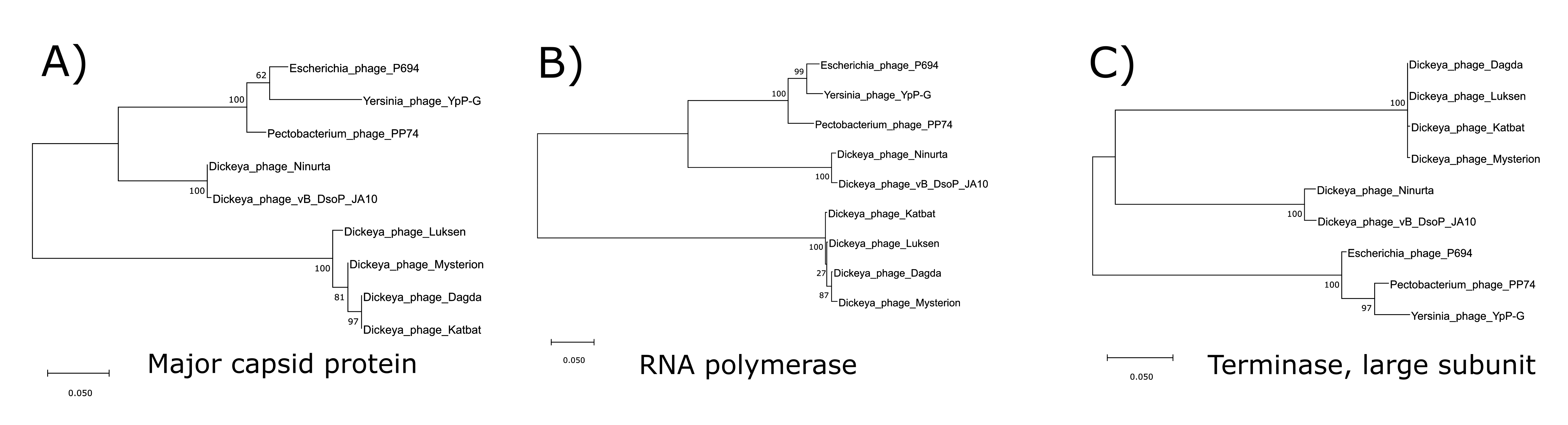

Supplement: Supplementary file 1 [file viruses-10-00621-s001.zip › sublementery/Figure_S2.png]

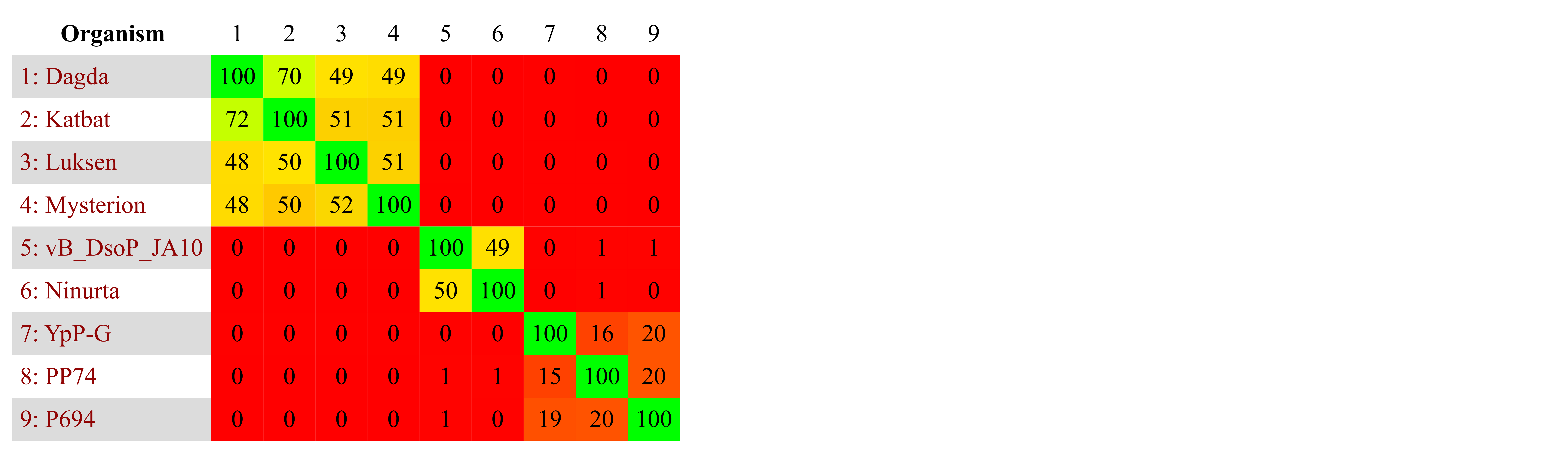

Supplement: Supplementary file 1 [file viruses-10-00621-s001.zip › sublementery/Figure_S3.png]

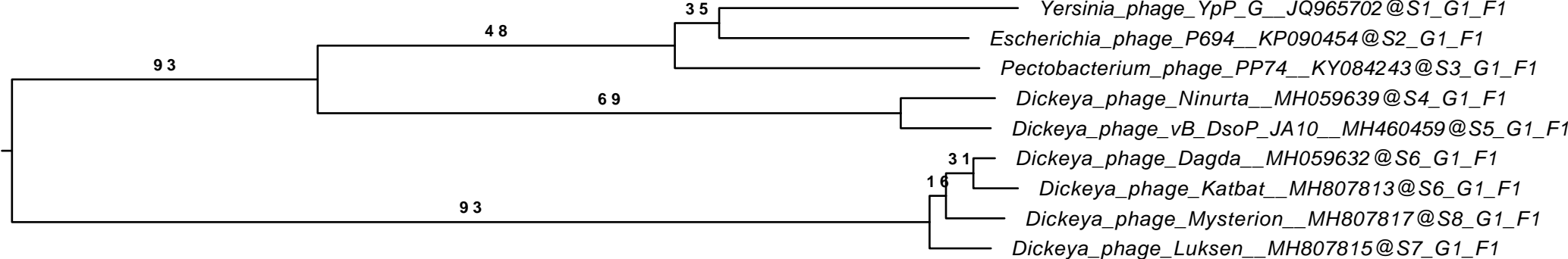

0.03

Supplement: Supplementary file 1 [file viruses-10-00621-s001.zip › sublementery/Figure_S4.pdf]
